# Small Molecule GSK-3 Inhibitors Safely Promote the Proliferation and Viability of Human Dental Pulp Stem Cells—In Vitro

**DOI:** 10.3390/biomedicines11020542

**Published:** 2023-02-13

**Authors:** Samer Hanna, Riham Aly, Ghada Nour Eldeen, Alberto Adanero Velasco, Ruth Pérez Alfayate

**Affiliations:** 1 Clinical Dentistry Department, Biomedical and Sciences Faculty, European University of Madrid, 28005 Madrid, Spain; 2 Basic Dental Science Department, Oral Medicine & Dentistry Research Institute, National Research Centre, Dokki, Giza 12622, Egypt; 3 Stem Cells Lab, Center of Excellence for Advanced Sciences, National Research Centre, Dokki, Giza 12622, Egypt; 4 Molecular Genetics & Enzymology Department, Human Genetic & Genome Research Institute, National Research Centre, Dokki, Giza 12622, Egypt

**Keywords:** CHIR99021, drug repurposing, GSK3 inhibitors, hDPSCs, tideglusib, *Wnt* pathway

## Abstract

Small molecules have demonstrated promising results as successful alternatives to growth factors. In this study, focus was drawn to CHIR99021 and tideglusib as GSK-3 inhibitors known for their anti-inflammatory and regenerative potential. The effect of both tideglusib and CHIR99021 on the proliferation, viability, and stemness of human dental pulp stem cells (hDPSCs) was investigated to assess their possible role in regenerative dentistry. Briefly, hDPSCs were isolated from sound premolars extracted for orthodontic purposes. Cytotoxicity and proliferation assessment were performed via cell counting kit-8 followed by flow cytometric analysis of apoptotic marker ANNEXIN V. The effect of both small molecules on the stemness of hDPSCs was analyzed by qRT-PCR. Both tideglusib and CHIR99021 were proven to be safe on hDPSCs. The tideglusib concentration that resulted in higher viable cells was 100 nM, while the concentration for CHIR99021 was 5 nM. Both small molecules successfully induced cellular proliferation and demonstrated minimal expression of ANNEXIN V, indicative of the absence of cellular apoptosis and further confirming their positive effect on proliferation. Finally, both small molecules enhanced stemness markers expression as evidenced by qRT-PCR, which, again, highlighted the positive effect of both tideglusib and CHIR99021 on safely promoting the proliferation of hDPSCs while maintaining their stemness.

## 1. Introduction

Small molecules (SM) can be defined as those with molecular weights less than 1 kDa. Small molecules are required to be at least soluble in the lipid bilayer to facilitate cellular uptake by simple diffusion [1]. Having those properties, small molecules have the ability to cross biological barriers, enter cells, and also access cell organelles [2]. Indeed, small molecules have shown promising results as successful alternatives to growth factors (GF) [3]. SM overcome most of the shortcomings of GF, since they are unlikely to induce an immune response and are considered protein stable [4]. Also, SM are considered cost effective and pose minimal risk of cross-species contamination [5].

An ongoing list of small molecules is commercially available, however, in this study, focus is drawn to CHIR99021 and tideglusib as GSK-3 inhibitors. GSK-3 inhibitors can be divided into two main categories: adenosine triphosphate (ATP)—competitive GSK-3 inhibitors and non-ATP-competitive inhibitors. ATP competitive GSK-3 inhibitors include different example of drugs such as pyrazolopyrimidines, benzimidazoles, pyridinones, and pyrimidines. On the other hand, the non-ATP-competitive inhibitors include thiadiazolidinones (TDZDs), 5-Imino-1,2,4-thiadiazoles (ITDZs), and tideglusib [6].

GSK-3 inhibitors were widely investigated in different clinical trials, as evidenced in the field of medicine literature. Using tideglusib to treat progressive supranuclear palsy cases was reported to be generally well-tolerated by the body, safe to use, with minimum side effects being mainly asymptomatic [7]. Also, tideglusib was effective in decreasing the progression of atrophy in the whole brain, especially the parietal and occipital lobes [8]. Likewise, its administration was recorded as safe with minimal side effects to the patients [7,9]. Tideglusib resulted in few adverse effects such as elevation of alanine amino transferase (ALT) [7]. In June 2017, the US Food and Drug Administration (FDA) and European Medicines Agency (EMA) has granted tideglusib the orphan drug status in the rare disease, tauopathy progressive supranuclear palsy (PSP) [10]. More recently, tideglusib was also granted approvals for treatment of myotonic dystrophy type 1 (DM1) [11].

CHIR99021, on the other hand, is considered one of the most selective inhibitors of GSK-3 that acts via ATP competitive inhibition [12]. It shows a 350-fold selectivity toward GSK-3β compared to cyclin-dependent kinases [13]. It is widely regarded as a *Wnt* signaling agonist that mainly functions through inhibiting the activity of GSK3b while maintaining the *Wnt* pathway in an active state [14]. Several studies conclude that CHIR 99021 improves the isolation efficiency of embryonic stem cells (ESCs) in both human and mice in addition to promotion of self-renewal [15]. CHIR 99021 regulates gene expression through increasing β-CATENIN cytoplasmic levels, which leads to prolonged interaction with the *Tcf* family of transcription factors together with nuclear translocation [16]. Microarray data demonstrate that CHIR99021 influences the *Wnt*/β-catenin pathway through stabilizing β-catenin, and also through modulating several other pluripotency-related signaling pathways such as TGF-β, Notch, and MAPK signaling pathways [17]. Collectively, both CHIR 98014 and CHIR 99021 are generally considered highly selective inhibitors of GSK-3 [13], and the specificity of CHIR 99021 was confirmed to be the most potent and specific inhibitor of GSK3 from within all tested GSK inhibitors [18,19]. Recently, CHIR99021 gained increased attention as a potent small molecule with potential application in dentistry. Heng et al., 2019 [20] tested the neurogenic differentiation effect of CHIR99021 and seven other small molecules over dental pulp stem cells (hDPSCs), stem cells from apical papilla (SCAPs), and gingival mesenchymal stem cells (GMSCs). This study confirmed the potential neurogenic differentiation effect of the eight small molecules on the dental stem cells, denoting the potential role this molecule could play in therapeutic applications.

In dentistry, the process of reparative dentin formation involves the activation of *Wnt*/β-cat signaling where Axin2 is a negative regulator and GSK3 is a major enzyme. In fact, various GSK3 inhibitors have illustrated an effect on promoting dentin repair. Over the past few years, there has been numerous trials to regenerate dentine in an attempt to substitute dental material and achieve an innate dentine repair. Previous studies investigating dentinogenesis demonstrated the role of *Wnt*/βcatenin signaling specifically as an important front runner in tissue regeneration and repair. In this context, GSK-3 inhibitors act as *Wnt* agonists that can effectively stimulate the *Wnt*/βcatenin pathway, especially given that they are connected to reparative dentin formation. Both tideglusib and CHIR99021 are potent cost-effective GSK-3 inhibitors small molecules that can act as signaling molecules to *Wnt*/βcatenin. However, to the best of our knowledge, only a few studies have investigated their role on the viability and proliferation of hDPSCs. We hypothesized that those two small molecules can elicit a regenerative effect if incorporated in vicinity to the dental pulp where dental pulp stem cells are normally present. Thus, the aim of the current study is to initially determine the cytotoxic effect of both tideglusib and CHIR99021 small molecules on hDPSCs and to investigate the effect of both molecules on the proliferation, viability, and stemness of human dental pulp stem cells.

## 2. Materials and Methods

The study was approved by the Ethical Committee of the Medical Research of the National Research Centre, Egypt (Ethical approval number: 5435062021).

### 2.1. Sample Collection and Isolation

The participants of the study were recruited at a private dental practice. The nature of the study was explained and consent was obtained from the participants of the study prior the start of the study. Anterior teeth were extracted from patient A (40 years old) for aesthetic/orthodontic reasons and sound upper central and lateral incisors were extracted from patient B (45 years old) for periodontal reasons. All collected teeth were vital, sound, and showed no signs of carries. Pulp extirpation was achieved using manual hand endodontic files and pulp tissues were transferred to the prepared media comprising (Dulbecco’s modified Eagle’s medium (DMEM), penicillin–streptomycin, 10% fetal bovine serum, and amphotericin B) in a cell culture dish. All procedures were carried out in a sterile laminar flow cabinet. Using a sterile tweezer and scalpel, pulp tissue was minced into small pieces. Minced pulp tissue was transferred into a new test tube (Corning, Corning, NY, USA). The sample was centrifuged using Sigma 6-16KS centrifuge (Sigma, Darmstadt, Germany) for 3 min with 30,000 rpm. Later, the old media was discarded and pulp tissue was subjected to digestion using Collagenase solution (Collagenase NB4 (Type 2), phosphate-buffered saline) for 30 min at 37 °C.

### 2.2. Identification and Characterization of Isolated DPSCs by Flow Cytometric for Surface Marker Expression

To validate the presence of MSCs in the isolated cells and rule out the presence of non-MSCs, flow cytometric analysis (FACS) for CD90, CD105, and CD34 was conducted. Cytomics FC500 flow cytometer (Beckman Coulter, Brea, CA, USA) and CXP software version 2.2 was used. The following steps were used to measure the expression of MSC markers: the adhering cells were detached and the cell density was adjusted to 1106 cells/mL. Then, at 4 °C in the dark, 1106 cells were incubated with 10 mL monoclonal antibodies CD34 to demonstrate hematopoietic negative, as well as CD90 and CD105 to demonstrate mesenchymal positivity (Beckman Coulter, USA). Isotypes were used as a control group. After a 20 min incubation period, 2 mL of PBS supplemented with 2% FBS was added to tubes containing monoclonal-treated cells. After centrifugation for 5 min at 2500 rpm, the supernatant was discarded and the cells were suspended in 500 mL PBS with 2% FBS.

### 2.3. Determination of the Least Cytotoxic Dose of CHIR99021 and Tideglusib on DPSCS

Samples were prepared for cell counting before the start of the cytotoxicity procedure. A total of 50 µL of suspended cells were mixed with 50 µL of trypan blue stain 0.4% and the sample was sent for counting with the Countess II FL Invitrogen (Thermo Fisher Scientific, Waltham, MA, USA). After calculations of the correct ratio of suspended cells versus media, 100 µL (2000 cells) was added to each well. After platting, cells were left to attach for 24 h, in an incubator at 37 °C. After 24 h, CHIR99021 and tideglusib were added at different concentrations: CHIR99021 5, 10, 15, 20, and 25 nM and tideglusib 10, 50, 100, 200, and 250 nM. Then, 10 μL of the specific drug was added to each well of the 96 well plate to stimulate the cells (two negative control wells containing cells were added with the same volume of culture medium without drugs).

The plate was then incubated at 37 °C, 5% CO_2_, and 100% humidity for the time period set for the cytotoxicity test 1 and 3 days. On day 1 and on day 3, 10 μL CCK-8 buffer was added and the plate was incubated at 37 °C, 5% CO_2_ in a cell incubator for 1~4 h as per manufacture instructions. Finally, the absorbance was measured at 450 nm using a microplate reader (Thermo Multiskan microplate reader).

### 2.4. Assessment of Cell Proliferation via CCK8 Assay

To assess the effect of both drugs on hDPSCs proliferation, the same procedures as cytotoxicity, using the enhanced cell counting kit 8, were followed. (WST-8/CCK8). Results were compared to the control group where hDPSCs were not subjected to either drugs. Cell number during proliferation testing was 5000 according to the CCK 8 manufacturer instructions. Proliferation testing was performed at 24 h, 48 h, and 72 h and represented in a proliferation curve.

### 2.5. Flow Cytometric Surface Marker Expression Analysis of Apoptotic Marker ANNEXIN V

The ANNEXIN V binding capability of treated cells in both groups was evaluated by flow cytometry using the ANNEXIN V FITC Detection Kit (BD Pharmingen, San Jose, CA, USA) according to the manufacturer’s procedure described for the detection of apoptosis.

### 2.6. Assessment of Stemness Properties by Real-Time Quantitative PCR

Gene expression analysis for stemness markers (OCT4, SOX, and NANOG) was performed to analyze the influence of both tideglusib and CHIR99021 on the stem cell characteristics of hDPSCs. The presence of such markers reveals stem cells’ ability to self-renew as well as their undifferentiated status [21]. Real-time quantitative polymerase chain reaction analysis differential expressions of the three stemness markers were carried out using the following primers: OCT-4, 5′-AACGACCATCTGCCGCTTTGA-3′ and 5′-CTCTCACTCGGTTCTCGATAC-3′; SOX2, 5′-ATCGAGCAGCTGACTACACTG-3′ and 5′-TGCGAGTAGGACATGCTGTAG-3′; NANOG, 5′-GAAGGAAGAGGAGAGACAGT-3′. Samples were run three times. The automatic cycle threshold (Ct) setting was used to analyze the data with the relative expression software tool (REST). The CT method was used to compute the relative expressions (REs) of the sample genes, with GAPDH serving as an internal control. Triplicate qRT-PCR assays were performed. The average values and standard error of the mean were used to present the data.

### 2.7. Statistical Analysis

Using Graphpad Prism version 7 software (©2016 GraphPad Software, Inc., San Diego, CA, USA), statistical tests, D’Agostino and Pearson, Shapiro–Wilk, and KS normality test were performed in order to check the normality distribution of the results. Following normality testing, results were presented and analyzed using the 1-way ANOVA non-parametric test Kruskal–Wallis test. Values of *p* < 0.05 were considered statistically significant.

## 3. Results

### 3.1. Isolation of hDPSCs

**Figure 1:**
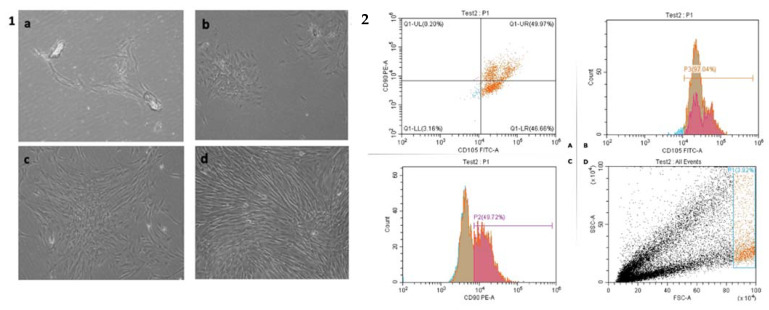
Photomicrograph illustrating DPSCs culture phases starting (**1**) from single cell attachment 3 days after isolation (a), colony formation (b), increase in colony size (c), until reaching confluence (d). (**2**) Phenotypic characterization of isolated DPSCs by flow cytometry showing the percentage of cells expressing CD105 and CD 90 in cells derived from pulp tissue after third passage in culture. (A) CD 105 (FITC fluorophore) versus CD 90(PE fluorophore) dot-plot showing the gate of the sample used for tracing the quadrants. Events observed beyond the lower left quadrant were considered positive. (B) Gate selection by size (FSC, forward scatter) and granularity (SSC, side scatter) of the cells. (D) Histograms showing individual data of frequency of CD105, CD90 of mesenchymal stem cells (MSCs) derived from dental pulp tissue. (C).

**Figure 2:**
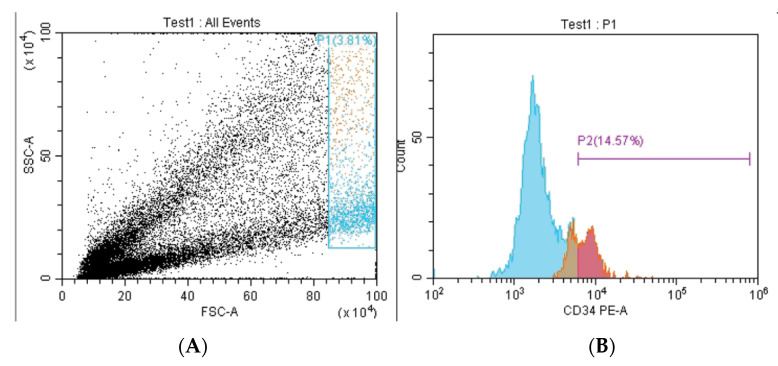
Phenotypic characterization of isolated DPSCs by flow cytometry showing the percentage of cells expressing CD34. (**A**) Gate selection by size (FSC, forward scatter) and granularity (SSC, side scatter) of the cells. (**B**) Histograms showing frequency of CD34 (PE fluorophore) perecntage of cells derived from dental pulp tissue.

In the current study, to confirm that the isolated DPSCs maintained their phenotypic characteristics after growth in culture, DPSCs were subjected to flow cytometry analysis. Stem cell identity of the DPSC was confirmed by positive expression of mesenchymal stem cell markers (CD 90–CD 105) (Figure 1) and negative expression of CD 34 (Figure 2).

### 3.2. Determination of the Least Cytotoxic Dose of CHIR99021 and Tideglusib

**Figure 3:**
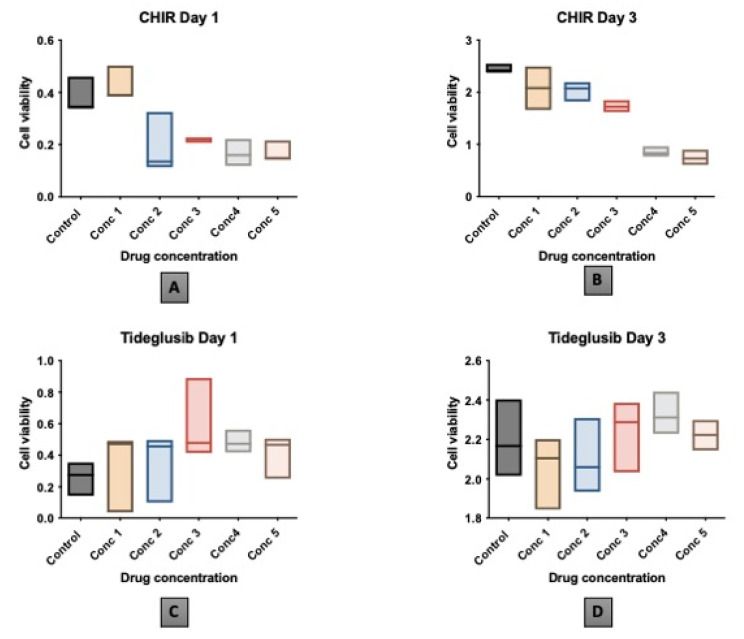
One-way ANOVA graph presentation of drug concentration (CHIR99021: 5, 10, 15, 20, 25 nM and tideglusib: 10, 50, 100, 200, 250 nM) vs. cell viability of: (**A**) CHIR99021 at day 1. (**B**) CHIR99021 at day 3. (**C**) Tideglusib at day 1. (**D**) Tideglusib at day 3.

Cytotoxicity was measured at different time intervals (24 h and 72 h) with different drug concentration (Conc). Conc. 5 (25 nM) of CHIR99021 results in a statistically significant lower viable cell count in comparison to the control group. However, no other statistical significance is registered between the different concentrations of each drug over the viability of the cells. (Table 1 and Table 2) (Figure 3). Yet, as in the CHIR99021 group, conc. 1 (5 nM) results in the highest viable cell count and in the tideglusib group, conc. 3 (100 nM) results in the highest viable cell count, therefore, both concentrations were the selected and applied to the following assessments.

### 3.3. Chir99021 and Tideglusib Promoted Proliferation and Cellular Viability

**Figure 4:**
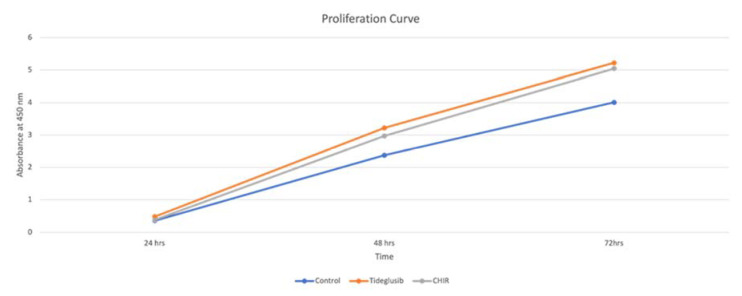
Graph presenting the proliferation curve of the three groups (Control–tideglusib–CHIR99021) over the period of 24, 48, and 72 h.

In the current study, it is noted that both CHIR99021 and tideglusib at the provided concentration increase cellular proliferation in comparison to the control group. (Figure 4).

**Figure 5:**
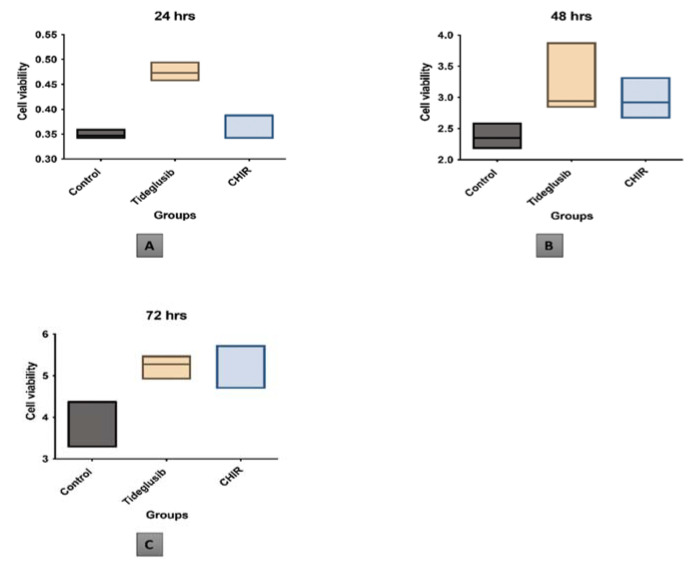
One-way ANOVA graph presentation of the three groups vs. cell viability of: (**A**) At 24 h. (**B**) At 48 h. (**C**) At 72 h.

However, no statistical significance is registered between the effect of the three groups over cell viabilities and proliferation. (Figure 5).

### 3.4. CHIR99021 and Tideglusib Protected hDPSCs from Apoptosis

**Figure 6:**
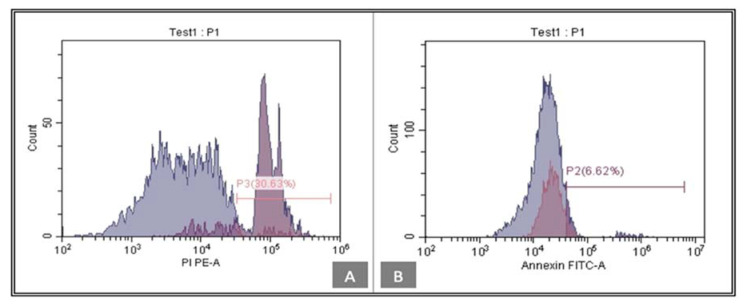
(**A**) Annexin-V analysis by flow cytometry was used to analyze apoptosis in tideglusib group. (**B**)The percentage of apoptotic cells was 6.62%. FITC, fluorescein isothiocyanate.

**Figure 7:**
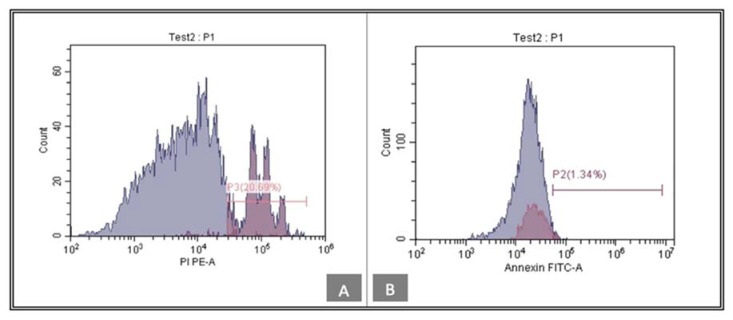
(**A**) Annexin-V analysis by flow cytometry was used to analyze apoptosis in CHIR99021 group. (**B**)The percentage of apoptotic cells was 1.34%. FITC, fluorescein isothiocyanate.

ANNEXIN V was tested in order to confirm cell proliferation according to the negative expression of the ANNEXIN V. Low levels of ANNEXIN V are noted within the tideglusib group, 6.62% (Figure 6), while the CHIR99021 group express a level of 1.34% (Figure 7).

### 3.5. CHIR99021 and Tideglusib Enhanced the Stemness Properties of hDPSCs

Three genes (OCT4, SOX2, NANOG) were PCR tested to confirm the maintenance of the stem cell identity after exposure to either CHIR99021 or tideglusib.

**Figure 8:**
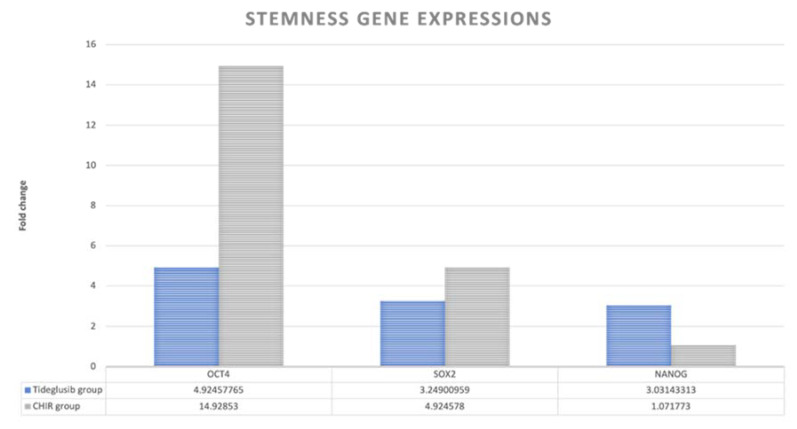
Chart presenting the different genes expression (OCT4, SOX2, and NANOG) within the study groups in relation to the control group (fold change).

In gene expression, the tideglusib group expresses the NANOG gene 3.031 times more than the control, while the CHIR99021 group expresses it 1.071 times in comparison with the control. Regarding the OCT4 and SOX2, the CHIR99021 group expresses those genes 14.928 and 4.924 times more than the control, respectively, while the tideglusib group expresses then 4.924 and 3.249 times more than the control, respectively (Figure 8).

**Figure 9:**
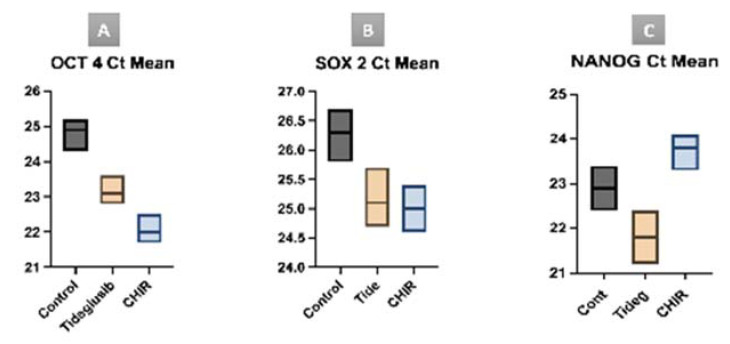
Graph presentation of the Ct mean of the different gene expression. (**A**) Ct mean OCT 4 gene expresses in higher number of cycles in control group than in tidesglub group and CHIR group. (**B**) Ct mean SOX 2 gene in higher number of cycles in control group than in tidesglub group and CHIR group. (**C**) Ct mean NANOG gene in higher number of cycles in control group than in tidesglub group and CHIR group.

Statistical analysis of the cycle threshold (Ct mean) of the gene expression within the study groups in comparison to the control group, results in the tideglusib group expressing the NANOG gene in statistically significantly fewer cycles in comparison to the CHIR99021 group (*p* = 0.0405). Also, the CHIR99021 group expresses the OCT4 gene in statistically significantly fewer cycles in comparison to the control group (*p* = 0.0219). Though it is noted that both study groups express the NANOG and OCT4 gene in fewer cycles, no other statistical significance is recorded between the groups. Also, similarly, both study groups express the SOX2 gene earlier than the control group, but with no statistical significance (Figure 9).

## 4. Discussion

In the current study, hDPSCs were chosen as the stem cell population, as it was our intentions to test the use and the effect of such drugs in conjunction with dental pulp as potential enhancers of vital pulp therapy and direct pulp capping material. The importance of this study as a first assessment of such drugs over the hDPSCs should be highlighted.

In the current study, in order to confirm the mesenchymal stem cell identity of the isolated cells, FACs analysis for mesenchymal stem cell surface marker was performed. The ability to express specific markers such as CD90, CD105, and CD73 while not expressing CD14, CD34, or CD45 is used to identify and confirm mesenchymal stem cells [22].

Dental stem cells are reported to be composed of 95% and more of CD29+, CD44+, CD73+, CD90+, and CD105+ cells, while less than 2% of dental stem cells express the panleukocyte marker CD45, the haematopoietic/endothelial cell marker CD34, the monocyte and macrophage markers CD11 and CD14, the B-cell marker CD79 and CD19, or HLA class II [23]. Also, the International Cellular Therapy Association developed minimum criteria for MSCs identification: that MSCs should express CD105, CD73, and CD90, and have no expression of CD45, CD34, CD14, and CD11b [24]. Interestingly, Bhandi et al., 2021 recently suggested the link between aging in dental pulp stem cells and the role of different endogenous molecules such as parathyroid hormone as a deciding factor in the therapeutic application of stem cells and to enhance the quality and efficacy of clinical grade stem cells [25].

In the current study, flow cytometry results are similar to that of previous studies, which confirms the positive expression of mesenchymal stem cell markers (CD 90–CD 105), and the negative expression of CD 34, which confirms the mesenchymal nature of the isolated DPSCs [26,27]. Before investigating the effects of CHIR99021 and tideglusib on hDPSCs, we first determined their cytotoxic effects on the cells. Two measurements points were set in the current study for the cytotoxicity testing where cellular responses were intended to be evaluated at 24 h and 72 h. Testing periods were set at these intervals according to ISO standards of the cell viability testing [28]. Similarly, Kook et al. used the same time intervals to test the effect of H_2_O_2_ over human periodontal ligament fibroblasts (HPLFs) [29]. Again, in a similar study that investigated MS-275, a histone deacetylase inhibitor, cytotoxicity on hDPSCs was tested after 72 h [30].

In the present investigation, cell counting kit 8 was used for the cytotoxicity analysis due to its superiority over other techniques. Testing the safest drug concentration of the CHIR99021 on hDPSCs after 24 h and 72 h shows that CHIR99021 at 25 nM results in significantly lower viable cells in comparison to the control. However, although no other statistical significance was recorded between the different concentrations of CHIR99021 and the control, CHIR99021 at concentration 5 nM results in the highest number of viable cells (mean: 2.079667 and SD: 0.340071). These results indicate that the higher the drug concentration administrated, the lower the count of cell viability received. Similar results are presented by Naujok et al., 2014, who compared the cytotoxicity of four different GSK-3 inhibitors drugs on mouse embryonic stem cells [19]. The results of this study concluded that the viability of the mouse embryonic stem cells was reduced with the increase in drug concentration when used at 2.5, 5, 7.5, and 10 nM [19]. Likewise, in another study assessing the effect of CHIR99021 on mice embryonic stem cells, their findings reveal that cells treated with higher or equal than 5 μM CHIR99021 demonstrate cell viability similar to that of control cells, and that cell viability is inhibited when CHIR99021 concentration is above 10 μM [17].

When we evaluate the concentration of tideglusib that yielded better cell viability after 72 h, no statistical significance is noted between different concentrations, whereas, 100 nM concentration illustrates the highest cell viability (mean: 2.236 SD: 0.148333). In a study conducted by Oncu et al., 2020, the authors tested the effect of tideglusib at different concentrations on human gingival fibroblast (hGF), periodontal ligament fibroblast (hPDLF), and osteoblast (hOB) to analyze the potential efficacy of tideglusib in periodontal regeneration. The authors concluded that 200 nM decreased the viability significantly in comparison to 50 nM when applied to the hPDLF [31]. Similar to those results, when tideglusib was administrated at different concentrations (200, 100, and 50 nM), the authors concluded that the best concentration to be used is the 50 nM [32].

In our study, although no statistical significance is noted between the three groups, it is important to note that the proliferation curve of the hDPSCs in the tideglusib and CHIR99021 groups is enhanced compared with that in the blank control group at day 3. Also, the curve of both study groups is very similar, indicating that both drugs have a similar effect on hDPSCs. Similar to our results, Oncu et al., 2020, also noted that tideglusib administered to human periodontal stem cells did not exert any statistically significant difference on the proliferation [31].

In the current study, we further confirmed the proliferation results through the analysis of the expression of ANNEXIN V, an early apoptotic marker. Evaluation of its expression indicates the possible apoptotic effect inflicted by either of the used small molecules on hDPSCs. According to Kanjevac et al. (2012), ANNEXIN V staining is a more effective tool for the identification of apoptosis at early stages rather than nuclear-changes-based assays such as DNA fragmentation [33]. ANNEXIN V expression is minimally detected by flow cytometry and its presence is considered low in both treatment groups tideglusib and CHIR99021 (6.62% and 1.34%, respectively). Both drugs do not show a negative effect on hDPSCs viability, which proves their safety to use in close relation to the pulpal tissue.

With the intention of testing the effect of both drugs on the stemness capacity of the hDPSCs, stemness markers were quantified using RT–PCR. OCT4, NANOG, and SOX2 are considered as self-renewal regulatory factors [34]. OCT4 is considered to be one of the Pit-Oct-Unc (POU) family of transcription factors [34]. OCT4 play a key role in preserving the pluripotent state of embryonic stem cells and is considered as an important stem cell marker, which is an essential transcription factor during human embryogenesis [35]. SOX2, a member of the SOX (SRY-related HMG box) gene family, is important for its action in controlling neural progenitor cells by prohibiting their ability to differentiate [36]. However, NANOG, a recently described gene, plays a critical role in regulating the cell fate of the pluripotent inner cell mass during embryonic development. NANOG is known to maintain the pluripotent epiblast and prevent differentiation to primitive endoderm [37]. It is also believed to work together with other important pluripotent factors such as OCT4 and SOX2 to control a set of target genes that have important functions in ES cell pluripotency [38,39]. Within the dental-related tissues, the human dental pulp stem cells (hDPSCs) and human dental follicle stem cells, those pluripotency markers were proven to be expressed [40].

In the current study, the expression of OCT4, NANOG, and SOX2 is confirmed by RT-PCR within the three groups (control, tideglusib, and CHIR99021). Our results are in conjunction with other studies where the expression of those genes is also confirmed in both hDPSCs [39]. Similar to other MSCs, in the current study it is confirmed that hDPSCs express pluripotency markers such as NANOG and OCT4 in all three study groups.

Our findings reveal that the expression of OCT4 in the CHIR99021 group is the highest compared to tideglusib and the control group having the least gene expression (fold change 14.928, 4.924, and 1, respectively). Also, for the SOX2 gene, the CHIR99021 group exhibits the highest results (fold change 4.924, 3.249, and 1, consecutively). However, for the NANOG gene, the tideglusib group expresses the highest gene expression between the three groups with (fold change 3.031), while the CHIR99021 and control group (fold change 1.071 and 1, respectively). These results both indicate and confirm that both treatment groups with the indicated doses have a positive effect on the stemness of the hDPSCs. Statistical analysis of the results was made with Kruskal–Wallis test. A *p*-value < 0.05 was considered statistically significant. Cycle threshold (CT mean) was recorded for each gene expression in each group. The tideglusib group expresses the NANOG gene in statistically significantly fewer cycles compared to the CHIR99021 group (*p* = 0.0405). This finding indicates that NANOG gene is present initially in higher concentration within the tideglusib group sample, confirming the positive effect of tideglusib on the stemness of the hDPSCs. Moreover, the CHIR99021 group expresses the OCT4 gene in statistically significantly fewer cycles in comparison to the control group (*p* = 0.0219). Though it is noted that both study groups express the NANOG, SOX2, and OCT4 genes in fewer cycles, no other statistical significance are recorded between the groups.

The limitations of the current study can be summarized as follows. ANNEXIN V testing was only performed for the study groups and not the control. This could be thought of as a limitation as no comparison was made between the study groups and the control, however, the importance of the test was to ensure that no apoptosis was initiated. As this is an in vitro study, testing drug effects on specific stem cells to check for vitality and ability to differentiate and proliferate, transferring this technique and theory to a higher evidence-based study such as a randomized clinical trial would confirm the study results and confirm the potential use of those drugs in the clinical settings. The current study highlights the positive effect of both small molecules tested on hDPSCs. With further investigations, the use of hDPSCs combined with small molecules could impact future research to translate into higher evidence-based studies. Such advancements to the field of regenerative dentistry could be ultimately translated, clinically enabling full dentin regeneration for direct pulp capping, which does not only save the tooth from unnecessary root canal treatment, but can also strengthen the tooth structure naturally while avoiding the application of any synthetic material.

## 5. Conclusions

In conclusion, both tideglusib and CHIR99021 in the provided concentrations are proven to be safe on hDPSCs. The tideglusib dose that results in higher viable cells is 100 nM, while the concentration for CHIR99021 is 5 nM. Both small molecules successfully induce cellular proliferation, which indicates that both of them are potent enhancers of proliferation. Also, both tideglusib and CHIR99021 demonstrate minimal expression of ANNEXIN V, indicative of the absence of cellular apoptosis and further confirming their positive effect on proliferation. Finally, both small molecules enhance stemness markers expression as evidenced by qRT-PCR, which, again, highlights the positive effect of both tideglusib and CHIR99021 on safely promoting the proliferation of hDPSCs while maintaining their stemness.

## Figures and Tables

**Table 1:** CHIR99021 different doses cytotoxicity over hDPSCs at day 3.

	Control	5 nM	10 nM	15 nM	20 nM	25 nM
Minimum	2.394	1.663	1.825	1.621	0.7660	0.6070
Median	2.398	2.080	2.076	1.727	0.8300	0.7290
Maximum	2.550	2.496	2.196	1.850	0.9630	0.8980
Lower 95% CI	2.226	1.045	1.562	1.448	0.6034	0.3817
Upper 95% CI	2.668	3.114	2.503	2.017	1.103	1.108

**Table 2:** Tideglusib different doses cytotoxicity over hDPSCs at day 3.

	Control	10 nM	50 nM	100 nM	200 nM	250 nM
Minimum	2.016	1.844	1.935	2.034	2.228	2.144
Median	2.167	2.105	2.060	2.288	2.312	2.223
Maximum	2.404	2.202	2.309	2.386	2.443	2.299
Lower 95% CI	1.710	1.590	1.628	1.785	2.059	2.029
Upper 95% CI	2.682	2.510	2.574	2.687	2.597	2.415

## Data Availability

Data available upon reasonable request to the corresponding author.
